# The power of *Drosophila* in modeling human disease mechanisms

**DOI:** 10.1242/dmm.049549

**Published:** 2022-03-29

**Authors:** Esther M. Verheyen

**Affiliations:** Department of Molecular Biology and Biochemistry, Centre for Cell Biology, Development and Disease, Simon Fraser University, 8888 University Drive, Burnaby, BC, Canada V5A 1S6

## Abstract

Six years ago, DMM launched a subject collection called ‘Drosophila as a Disease Model’. This collection features Review-type articles and original research that highlight the power of *Drosophila* research in many aspects of human disease modeling. In the ensuing years, *Drosophila* research has further expanded to capitalize on genome editing, development of resources, and further interest in studying rare disease mechanisms. In the current issue of DMM, we again highlight the versatility, breadth, and scope of *Drosophila* research in human disease modeling and translational medicine. While many researchers have embraced the power of the fly, many more could still be encouraged to appreciate the strengths of *Drosophila* and how such research can integrate across species in a multi-pronged approach. Only when we truly acknowledge that all models contribute to our understanding of human biology, can we take advantage of the scope of current research endeavors.

## Abstract (precis)

**Summary:** This Editorial encourages us to embrace the power of the fly in studying human disease and highlights how *Drosophila* studies can be integrated with research in other species to further our understanding of human biology.

For over a century, scientists have used the fruit fly to learn about fundamental and evolutionarily conserved genetic and cellular processes. The pioneering work of Thomas Hunt Morgan and his students, in the early 20th century, proved that genes are located on chromosomes and led to the first chromosome linkage maps (Morgan, 1910). In the 1980s, Ed Lewis, Christiane Nüsslein-Volhard and Eric Wieschaus showed that individual genes could be mutated to cause characteristic embryonic patterning defects (Lewis, 1978; Nüsslein-Vollhard and Wieschaus, 1980). Their genetic studies allowed them to order genes within functional pathways through epistasis analyses. The genes they identified have counterparts across species and play key roles in development and disease from flies to humans. Indeed, much of the molecular circuitry for key signaling pathways, such as RAS, Notch, Hedgehog and Wnt, was elucidated in *Drosophila* (Ashton-Beaucage and Therrien, 2017; Bejsovec, 2018; Ingham, 2018; Salazar and Yamamoto, 2018). This rich history has established *Drosophila* as a powerful tool in biology, paving the way for further advances in basic and translational research.

## Disease modeling

**Fig. 1.**
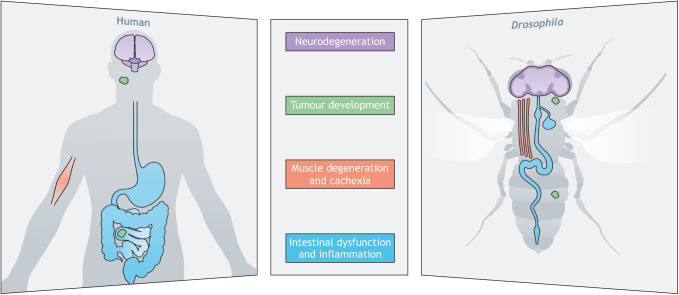
**Modeling of diverse diseases in the fly.** *Drosophila* can mirror many human diseases and disorders, including those associated with neurodegeneration, tumour development, muscle degeneration, and intestinal dysfunction and inflammation.

*Drosophila* has been used for decades to carry out basic research on developmental signaling pathways and to reveal molecular functions of human disease-associated genes (Ugur et al., 2016). About 75% of all human genes implicated in disease have functional homologs in *Drosophila* (Rubin et al., 2000). Owing to their low genetic redundancy, flies can be used to address specific questions about human disease that have been difficult to resolve in cell culture or in vertebrates (Bier, 2005), and many of the cell behaviors observed in normal and diseased cells can easily be modeled in the fly (Fig. 1). With the expansion of disease modeling into non-rodent models, such as zebrafish, *Drosophila* and *C. elegans*, a greater appreciation has arisen of how these models collectively contribute to our understanding of disease pathogenesis and treatment. In 2005, Ethan Bier highlighted the numerous disease categories that could be modelled effectively in *Drosophila*, from developmental disorders to neurodegeneration and cancer (Bier, 2005). To continue advancing these areas of investigation, researchers require sustained support from community resources they rely heavily on, such as stock centers and databases (see Box 1).

**Box 1. Community resources at risk**

A valuable feature of the fly community is its openness to sharing reagents and ideas. This applies to individuals who make their fly stocks freely available after publication and goes up to the fundamentally important stock centers spread across continents. Model organism databases are another essential resource, with the international *Drosophila* community being supported by FlyBase, one of the first databases to compile the genetic history and phenotypic characterization of mutant fly strains. More recent additions connect human genetics researchers to their *Drosophila*-focused colleagues to expand the accessibility of this resource (Millburn et al., 2016). FlyBase serves as an essential part of the *Drosophila* community and its existence, together with the other model organism databases (MODs), is at risk as funding agencies seek to consolidate resources. These issues were well described in recent opinion pieces (Bellen et al., 2021; Kling, 2021), and are highlighted in this issue in the interview with Hugo Bellen (Bellen, 2022). It is short-sighted to cut the funding for MODs because the integration of studies from numerous model systems will accelerate our understanding of gene function, protein networks and disease mechanisms.

*Drosophila* have proven particularly effective in studying cancer development and interactions between tumor suppressors and oncogenes (Villegas, 2019). Once it had been established that *Drosophila* tumors display many or most of the features found in human cancers, flies became an accepted system in which to rapidly dissect molecular mechanisms of tumorigenesis (St. John and Xu, 1997). Notably, this includes systemic wasting (cachexia) – a poorly understood clinical feature of many cancers – as reviewed in this issue by Norbert Perrimon and colleagues (Liu et al., 2022) and in a recent Review from David Bilder's group (Bilder et al., 2021). Furthermore, fly cancer models reveal heterogeneity in metabolic reprogramming depending on mutation load, which may provide more insight for studying human tumors (Wong and Verheyen, 2021). *Drosophila* also provide a powerful opportunity to model multimorbidity, such as cancer accompanied by obesity or aging, in a simplified genetic system (Choutka et al., 2022). Also in this issue, Julia Cordero and colleagues discuss the complex interplay between intestinal dysfunction and systemic disease, noting another area in which *Drosophila* studies can shed light on human medical challenges (Medina et al., 2022). These approaches all highlight a common theme of the power and versatility of *Drosophila* to study aspects of metabolism.

*Drosophila* genetic interaction and cell biological studies have shed light on underlying molecular mechanisms of neurodegeneration, such as Alzheimer's, Huntington's and Parkinson's diseases (Bolus et al., 2020). Indeed, novel genes implicated in such diseases can be uncovered through analyses in flies (Deal and Yamamoto, 2019), as has recently been shown for the p21-activated kinase 4 (PAK4; Mbt in *Drosophila*) (Pütz et al., 2021). PAKs have been implicated in Parkinson's disease and, in the latter study, Pütz et al. found Mbt to be required in specific neurons to prevent age-dependent loss of locomotor activity. Furthermore, patient-specific disease mutations can be modelled in flies to reveal mechanisms of pathogenicity (Lin et al., 2020), which is often more achievable in *Drosophila* as compared to mammalian models. For example, novel mutations in the *UQCRC1* gene have been implicated in familial Parkinson's disease, and assays in human cell lines, mouse models and *Drosophila* have assessed the pathogenicity of these variants. Fly models allowed rapid identification of age-dependent locomotor defects and loss of dopaminergic neuronal populations, as well as identification of neuromuscular junction defects. These studies were consistent with analyses in mouse models, highlighting the conserved functions and the power of *Drosophila* for rapid and physiologically relevant characterization.

## Rare disease studies in the fly

With advances in genome and exome sequencing, increasing numbers of patient variants are being identified. A challenge of rare disease studies is the time and cost of developing vertebrate models to establish pathogenic variant function. The fly offers a robust, rapid and physiologically relevant system in which to determine functions of mutant proteins (Wangler et al., 2017). Such studies are being driven in part through the actions of clinicians and model organism researchers. Leaders in these fields have successfully developed valuable networks and tools, such as Model organism Aggregated Resources for Rare Variant ExpLoration (MARRVEL) (Wang et al., 2017), the Undiagnosed Diseases Network (UDN) (Baldridge et al., 2021) and Canada's Rare Diseases Models and Mechanisms (RDMM) Network (Boycott et al., 2020). These groups bring together clinicians who identify patient mutations and seek to understand underlying causes of disease, with experienced model system researchers who can capitalize on *Drosophila* genetics and established phenotypes to rapidly provide functional insight into patient variants.

Numerous recent studies highlight the strength of the fly to model disease mechanisms and variant functionalization. For example, dominantly inherited limb–girdle muscular dystrophy type D2 (LGMDD2) causes progressive muscle degeneration owing to a single nucleotide deletion in the *transportin 3 (TNPO3)* gene, which removes a stop codon and results in a C-terminal extension to generate a protein of unknown function (Melià et al., 2013). Blázquez-Bernal and colleagues developed a fly model expressing mutant TNPO3, which recapitulated many of the clinical features (Blázquez-Bernal et al., 2021). In this model, the authors observed improvement of muscle degeneration and flight behavior upon treatment with chloroquine (an established autophagy blocker), suggesting that increased autophagy in patients contributes to muscle degeneration. This finding revealed that the defects are reversible, providing much needed insight into mechanisms of LGMDD2 pathogenesis and validating the use of *Drosophila* as a drug screening platform. In a different approach, Bangi et al. developed a personalized therapy for a patient with adenoid cystic carcinoma (ACC) by using a *Drosophila* model (Bangi et al., 2021). They generated a patient-specific fly strain [avatar] harboring five variants found in a patient with ACC. By using an iterative screening process, Bangi and colleagues were able to identify a three-drug cocktail that rescued transgene-mediated lethality, leading to a stable disease state in the fly. These drugs were then administered to the patient, who had failed to respond to standard-of-care treatments and, impressively, they provided the patient 12 months of stable disease before treatment resistance occurred. This approach uses a whole-animal model to provide a personalized screening platform that expands therapeutic options for treatment-resistant patients.

## Conclusion

*Drosophila* has played an essential role in our understanding of organismal development and tissue patterning. Taking advantage of this wealth of knowledge, researchers can leverage functional assays and genetic interactions in the fly to understand molecular mechanisms of disease. Future use of fly models for both common and rare diseases is likely to yield important insights into cellular functions and therapeutics. *Drosophila* continues to be an important tool in understanding fundamental biological principals and their applications to human medicine. DMM will continue to support and promote disease research in this powerful model organism, by publishing high-quality research and cutting-edge resource articles. DMM also encourages integration with other laboratory systems to potentiate translational research and aims to support all communities working towards this.
